# Psychosocial Modulators of Motor Learning in Parkinson’s Disease

**DOI:** 10.3389/fnhum.2016.00074

**Published:** 2016-02-29

**Authors:** Petra Zemankova, Ovidiu Lungu, Martin Bares

**Affiliations:** ^1^First Department of Neurology, Faculty of Medicine of the Masaryk University and St. Anne’s University HospitalBrno, Czech Republic; ^2^Behavioural and Social Neuroscience Research Group, Central European Institute of Technology-Masaryk UniversityBrno, Czech Republic; ^3^Department of Psychiatry, University of Montreal and Centre de recherche de l’Institut Universitaire de Gériatrie de MontrealMontreal, QC, Canada; ^4^Department of Neurology, University of MinnesotaMinneapolis, MN, USA

**Keywords:** Parkinson’s disease, motor learning, self-efficacy, task engagement, emotions, social interaction

## Abstract

Using the remarkable overlap between brain circuits affected in Parkinson’s disease (PD) and those underlying motor sequence learning, we may improve the effectiveness of motor rehabilitation interventions by identifying motor learning facilitators in PD. For instance, additional sensory stimulation and task cueing enhanced motor learning in people with PD, whereas exercising using musical rhythms or console computer games improved gait and balance, and reduced some motor symptoms, in addition to increasing task enjoyment. Yet, despite these advances, important knowledge gaps remain. Most studies investigating motor learning in PD used laboratory-specific tasks and equipment, with little resemblance to real life situations. Thus, it is unknown whether similar results could be achieved in more ecological setups and whether individual’s task engagement could further improve motor learning capacity. Moreover, the role of social interaction in motor skill learning process has not yet been investigated in PD and the role of mind-set and self-regulatory mechanisms have been sporadically examined. Here, we review evidence suggesting that these psychosocial factors may be important modulators of motor learning in PD. We propose their incorporation in future research, given that it could lead to development of improved non-pharmacological interventions aimed to preserve or restore motor function in PD.

## Introduction

In Parkinson’s disease (PD) research much effort is devoted nowadays to the development of complementary, non-pharmacological interventions, which could help alleviate the symptoms and slow down the neurodegenerative progression of the disease. Physical exercise and motor training have the potential to be such alternative interventions (Fisher et al., 2008; Goodwin et al., 2008), yet, their success essentially depends on individual’s capacity to acquire new motor skills, which is also affected by the disease. Finding new ways to boost motor learning capacity in the course of intervention will not only increase the efficacy of exercising and motor training in people with PD, but, more importantly, it will increase the likelihood of a successful intervention.

There is much evidence indicating that motor learning and performance can be improved in PD via additional sensory stimulation (visual or rhythmical) and task cueing, most likely through increased activity in basal ganglia and the cortico-striatal circuits (Nieuwboer et al., 2009; de Bruin et al., 2010). Yet, despite these advances, several important knowledge gaps remain. (1) Most studies investigating motor learning in PD used laboratory-specific tasks and equipment, with little resemblance to real life situations. Thus, it is unknown whether optimal stimulation and task cueing can be achieved in more ecological setups and whether individual’s task engagement can further improve motor learning capacity; (2) The role of social interaction in the process of skill learning in PD has not yet been investigated; (3) Despite evidence that individual’s mind-set, such as self-efficacy, strongly impacts performance and learning capacity (Mak and Pang, 2008; Salanova et al., 2011; Wulf et al., 2012), this issue has been sporadically addressed in PD research; and (4) In real-life, motor learning involves not only task-related motor-cognitive processes, but also requires managing task-related emotions, which have also motivational consequences; thus, self-regulatory mechanisms could play a crucial role in motor learning process, especially in PD, which is characterized by motor and emotional dysfunction.

In the current review, we will first discuss the evidence for the potential benefits of developing new ecological approaches in motor learning research in PD, with special focus on the role of social context as external modulators. Then, we will analyze the role of psychosocial factors as internal modulators of motor learning capacity, specifically patient’s self-efficacy and emotional state. Finally, we will link these findings with the context of PD neuropathology and potential for motor treatment regimes.

## The Underlying Link Between PD Neuropathology and Neuronal Correlates of Motor Sequence Learning

**Figure 1:**
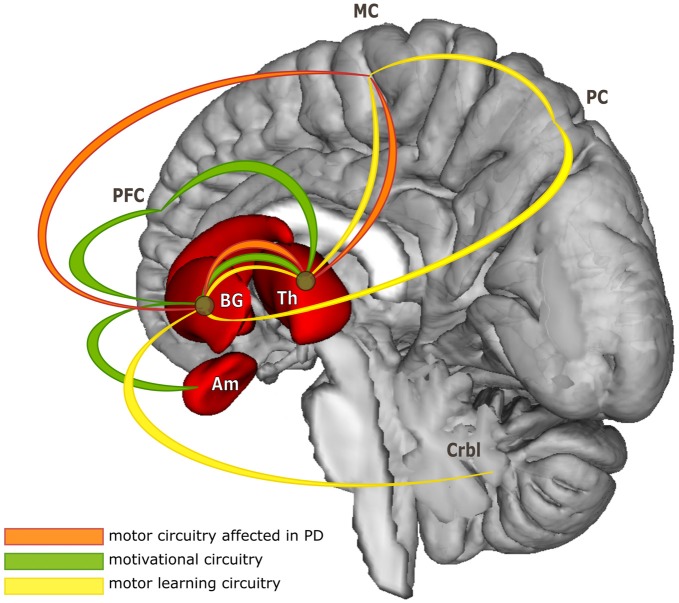
**Simplified model of functional circuitries of basal ganglia.** The figure illustrates considerable overlap of neuronal pathways subserving three different functions targeted in the review. For more detailed model of cortico-striatal and cortico-cerebellar systems contributing to motor skill learning see for example Doyon et al. (2003); for basal ganglia functional organization in Parkinson’s disease (PD) see Blandini et al. (2000) or Obeso et al. (2008); for basal ganglia motivational loop see Ikemoto et al. (2015). Abbreviations: BG, basal ganglia; Th, thalamus; Am, amygdala; PFC, prefrontal cortex; MC, motor cortex; PC, parietal cortex; Crbl, cerebellum.

PD is a progressive neurodegenerative disorder, characterized primarily by motor symptoms including tremor, rigidity, slowness of movement (bradykinesia) and gait difficulties. Typical PD patients present not only nigrostriatal dopaminergic cell loss in the basal ganglia, but also disruptions in mesocortical dopaminergic, noradrenergic, and other systems (Jellinger, 2012). These affect motor program selection by the striatal circuitry, with widespread effects in the entire cortico-striatal system (Amano et al., 2013). Much evidence from motor learning research indicates that acquisition of new motor sequences is based on increased neuronal activity in the cortico-striatal and cortico-cerebellar circuits and on a dynamic interaction between them (Doyon et al., 2009). In fact, striatum is involved in all stages of motor sequence learning with different parts of it deemed essential in each stage (Doyon and Benali, 2005). This indicates a remarkable overlap between PD neuropathology and neuronal correlates of motor sequence learning (see Figure 1).

Given this overlap and the fact that cerebral plasticity is maintained or increased through repeated practice and enhanced stimulation from the environment (Hultsch et al., 1999; Vance et al., 2010), it is conceivable that practicing or learning motor sequences in a rich and stimulating context may increase the effectiveness of non-pharmacological interventions aimed to preserve or restore motor function in Parkinson’s.

## Value of Ecological Experimental Setups in PD Motor Learning Research

A review of motor learning literature (Nieuwboer et al., 2009) indicates a relatively preserved acquisition and retention of motor skills in people with PD, despite reduced learning rates and efficiency as compared to controls. However, using additional sensory information and visual task cueing can optimize motor learning in PD with long-lasting effects (Nieuwboer et al., 2009; Sacrey et al., 2009; Anzak et al., 2011). For instance, some investigators have shown that the use of music as an external sensory cue helped performance in motor tasks in PD (McIntosh et al., 1997; Bernatzky et al., 2004; Sacrey et al., 2009; de Dreu et al., 2012). Importantly, patients benefited from rhythmical stimulation not only in terms of gait and postural control, but also in generating more complex motor sequences using upper limbs (Thaut and Abiru, 2010).

This research advanced significantly our knowledge about motor learning in PD, but the very fact that it was done mostly in controlled laboratory or clinical settings is, simultaneously, an asset and an important limitation, which may hamper its translation into real-life situations. While the setups used in these studies allowed for precise measurements and a good control of variables, they were also removed from the ecological context in which most daily living activities take place (i.e., at home, at work). For instance, implicit motor sequence learning is typically studied using the serial reaction time task (SRTT), in which participants have to respond to sequential or random cues by pressing buttons as fast and as accurate as possible (Muslimovic et al., 2007), a goal which is not ecological and may even be disengaging. In contrast, in real life, people learn sequences of key presses on a new phone, for example, while writing messages or playing games; thus the goal is to write or play, not to learn key sequences *per se*. In this context, learning the sequences of key presses represent the means by which an ecological goal is achieved. In addition, it is likely that PD patients are more susceptible to fatigue, anxiety, are less motivated and self-confident than their healthy peers, due to the compromised dopamine pathways. Therefore, it is possible that the laboratory setting might actually undermine their potential and the results would underestimate their actual motor learning capacity.

The above mentioned factors, such as the overall task engagement, reflecting individual’s level of arousal, interest and energy put into a given task (Salanova et al., 2011), have rarely been measured in PD motor learning studies. One notable exception is the research investigating the impact of console gaming technology. A systematic review provides evidence that exercising using console videogames improved not only the motor performance, but also task engagement in people with PD (Barry et al., 2014). In fact, a randomized controlled trial showed that the benefits of console game exercising on balance, cognition and some motor symptoms were as good as the typical rehabilitation regimen in PD and lasted for up to 60 days post-intervention (Pompeu et al., 2012). Altogether, these results suggest that exercising in a more ecological context, with complex sensory stimulation and meaningful goals might increase task engagement, and consequently boost the effects of motor rehabilitation and non-pharmacological interventions in PD (Nieuwboer et al., 2009; Nombela et al., 2013; Barry et al., 2014).

Regarding the possible action mechanisms by which enriched environment may enhance learning and memory in PD, a study with rodents showed that the observed functional changes were also associated with numerous neuronal changes, including altered cortical weight and thickness or increased dendritic branching and synaptic strength (Nithianantharajah and Hannan, 2006). These findings suggest a neurophysiologic mechanism by which external sensory stimulation may facilitate the signal neurotransmission in the impaired cortico-striatal loop, thus improving some of the Parkinsonian motor symptoms, the gait, as well as the execution of movements that were previously automatic. However, given the lack of PD research employing ecological experimental setups, it is yet unknown to what extent these methods could also improve motor learning capacity and whether the underlying mechanisms of action are based on the above mentioned types of neuronal changes.

## Social interactions and Motor Skill Learning in Healthy Subjects and PD Patients

Another important aspect, largely ignored in motor learning research in general, and in PD in particular, is the fact that in everyday life people usually learn and perform motor actions together with others (i.e., sports, dance, etc.) and not in isolation. However, the vast majority of motor learning literature is based on tasks presented to single participants in settings removed from natural physical and social environments. In a recent study (Lungu and Debas, 2013), researchers increased the ecological value of the SRTT by manipulating the social context (i.e., doing the task in solitude vs. together with a partner). The results showed that cooperation with a partner boosted motor performance as compared to the solitude condition, suggesting that social interaction can influence motor learning capacity.

Although only a behavioral study, the above-mentioned findings have relevant bearings on the neural mechanism mediating the effects of social context on motor learning. For instance, performing a motor task in an ecological setup, in a familiar social context (e.g., in collaboration with a friend), can be stimulating and provide a social reward to the individual. In this context, it has been shown that social rewards activate brain regions similar to those activated in response to monetary rewards, importantly involving the striatum (Izuma et al., 2008, 2010; Bhanji and Delgado, 2014). Rewards are generally associated with increased dopaminergic activity in the cortico-striatal system, which is known to play a key role in motor (sequence) skill learning and motor memory consolidation and automatization (Doyon, 2008; Yin et al., 2009; Debas et al., 2010; Sommer et al., 2014). In this context, it is noteworthy to mention that a study in which monetary values were used as reward (gains) or punishment (loses) in a procedural learning task indicated that reward improved motor learning through increased striatal activation, whereas punishment augmented motor performance (i.e., speed of execution), but not implicit sequence learning, through increased insular activation (Wachter et al., 2009).

Rewards stemming from social context (i.e., social interactions) can be seen as online modulators of motor learning and performance. Although in human research the rewards are typically operationalized as monetary incentives, there is evidence that social context can provide a benefit, above and beyond that of monetary reward itself. For instance, in the context of monetary games, earning a reward by cooperating with a partner evoked greater activity in ventral striatum as compared to gaining equivalent rewards in non-social condition (i.e., playing alone or with a computer partner), in addition to the fact that the mutual cooperation was valued as most satisfying by the participants (Rilling et al., 2002). In the motor domain, Sugawara et al. (2012) were the first to demonstrate the effect of social reward in form of praises given during initial training on offline motor skill consolidation, seen the next day, after a night of sleep. They showed that even when controlling for fatigue, alertness, duration and quality of sleep, the rate of offline improvements in motor sequence retention test were significantly higher in the “praised group” compared to individuals from the other two control groups, who received no self-related social feedback (Sugawara et al., 2012).

There is considerable overlap between the dopaminergic circuitry and the neuronal substrates affected by PD and those involved in reward-related information processing (Graef et al., 2010; van Wouwe et al., 2012; Balasubramani et al., 2015; see Figure 1). In some cases, PD patients are apathetic (Lawrence et al., 2011; Jordan et al., 2013), or have high level of impulsivity (Housden et al., 2010; Antonelli et al., 2014; Aracil-Bolaños and Strafella, 2016) often leading to depression or gambling addictions, respectively. Given that rewards, monetary or social, can be used to improve motor learning capacity in healthy individuals, it is imperative to investigate a similar approach in PD, too. There is evidence that despite impairments in dopaminergic circuitry involving striatum, PD patients possess compensatory mechanisms based on cerebellar and prefrontal cortex networks when processing rewards or feedback (Goerendt et al., 2004; Keitz et al., 2008). These mechanisms can be then used to modulate motor performance, as indicated by a study in which monetary rewards were found to speed up movement initiation and execution in PD patients with bradykinesia (Kojovic et al., 2014). However, there is a scarcity of research investigating facilitatory effects of rewards on restoring or improving motor learning capacity in PD and no study to assess the extent to which these effects can be elicited by rewards provided by the social context (i.e., social interaction). A better understanding of the effects of social rewards on motor skill acquisition and consolidation could lead to development of new intervention protocols that will incorporate and use social interactions to restore or improve motor functions and alleviate motor symptoms in PD. In line with this idea, we are currently conducting a study in which we aim to show that a console videogame with musical rhythms (Frets on Fire, a console videogame very similar to Guitar Hero), played in solitude or with a partner, will increase PD patients’ engagement in the task, which, in turn, will increase fine motor coordination in the upper limb and motor sequence learning capacity. By using a more ecological design than the one typically employed in most motor sequence learning studies, we hope to demonstrate that people with PD can have many benefits from playing this type of low-cost and safe videogame. They can improve their motor learning skills and the hand motor coordination while finding pleasure in so doing, in addition to increasing the interaction with family and friends. In long run, if we can demonstrate that this type of gaming with a social component can preserve motor functions, we may employ it to slow down Parkinson’s progression, the need for more medication and exposure to adverse side effects.

## Mindset and Motor Learning in PD

People’s beliefs in their capabilities to produce given attainments are a psychological construct commonly known as self-efficacy (Bandura, 2001, 2004). High levels of self-efficacy were found to be consistently associated with increased performance in a variety of tasks in healthy individuals (Bandura, 2001, 2004; Clair et al., 2005; McAuley et al., 2006; Salanova et al., 2011). Unfortunately, this concept has not been fully integrated in PD research despite encouraging, but limited evidence. For instance, Mak and Pang (2008) were the first to demonstrate in PD patients that balance self-efficacy was an important determinant of their walking abilities. This findings suggests that increasing patients’ confidence in their own skills, may have positive consequences on their motor function. Conversely, individuals’ lack of confidence in their capabilities (i.e., low self-efficacy), may increase their psychological stress and anxiety while being engaged in motor activities, in turn having a detrimental effect on their performance. Yet, the extent to which self-efficacy can influence motor learning capacity in PD and its mechanisms of action remain unknown to date.

In addition to external factors that can impact motor skill learning, the individuals’ internal mind set (i.e., the attitudes and beliefs with which a person approaches the task) may also play a role (Jourden et al., 1991). In particular, self-stereotypes or assumptions about loss of abilities may contribute to further decline in performance (Levy, 2003). This aspect could be especially relevant in people with PD who experienced visible losses in their motor skills, which may lead to an underestimation of their real, available capacity. The message transmitted to the public by the research community, based on the fact that most studies on motor learning in PD associate the deficit with the disease and neurodegeneration, serves only to reinforce patients’ expectation they should perform worse in these tasks. Thus, a vicious circle may form where PD patients become more anxious, feel under stress and less confident about their existing motor learning capacity when it comes to learning a new motor skill, which, in turn, will only hamper their learning process and performance. This is not to say that neurodegeneration is not real and it will not affect motor learning capacity in an objective manner, but just as the negative impact of neurodegeneration may be alleviated by medication, there is evidence that motor learning capacity may also be enhanced by breaking the negative self-beliefs and improving self-efficacy (Jourden et al., 1991; Mak and Pang, 2008; Barry et al., 2014).

There are two main approaches employed in interventions aimed to boost self-efficacy: providing individuals with a better sense of control over the task at hand through instructions or task setups and reinforcing the self-confidence through positive feedback on performance. In the context of the first approach, there is evidence that providing enhanced expectancies and support of autonomy during learning process in healthy individuals increased self-efficacy and these two factors were found to have an independent effect on learning (Hooyman et al., 2014; Wulf et al., 2014). In PD patients, it has been demonstrated that self-controlled practice enhanced not only individuals’ self-efficacy, but also their motor performance and learning (Chiviacowsky et al., 2012). Specifically, PD patients in an experimental group, who had the choice to use or not a balance pole when learning a balance task (i.e., increased sense of control), experienced lower levels of nervousness and were less concerned about their body movements than patients in a control group whose use of the pole was yoked to the experimental group. In addition, the experimental group learned the task better than the control group (Chiviacowsky et al., 2012). The authors interpreted these results to indicate that learner-controlled practice plays not only a motivational function, but it may fulfill the basic psychological need for autonomy, which may be more important in people with PD than in their healthy counterparts.

The second approach in boosting self-efficacy is through the use of performance feedback, which can be provided by the social context. In real life, people usually learn skills in social contexts and interacting with others; as such, they tend to compare with others either explicitly or implicitly. For learners, the normative feedback (i.e., how other people perform in the same task) seems to be important; yet, only few studies have investigated, so far, the impact of social-comparative feedback on self-efficacy and motor learning capacity. For instance, positive social-comparative feedback was shown to increase performance in the retention test in a novel motor task in children (Ávila et al., 2012). In addition, individuals in the positive social-comparative feedback perceived themselves as being more competent as compared to the group with no social feedback. A similar effect was demonstrated in younger and older adults, where experimenters used positive social comparison to manipulate individuals’ perceived competence (i.e., self-efficacy) when learning a novel balance task (Lewthwaite and Wulf, 2010; Wulf et al., 2012). The authors reported that positive social comparison not only decreased individuals’ level of nervousness and concerns about performance during learning, but it had also a long-term impact on motor learning, as revealed by increased performance in the delayed retention test, when social feedback was no longer provided (Lewthwaite and Wulf, 2010; Wulf et al., 2012).

Given the power of the mindset to modulate, either positively or negatively, motor learning and performance, the scarcity of research investigating its effects in PD is peculiar and constitutes an important knowledge gap to be addressed by future studies.

## The Role of Emotions in Motor Learning

A common factor underlying the facilitatory motivational effects on motor performance across the various domains we described above (task engagement, social interactions, self-efficacy) are the emotions associated with the learning process. During motor learning, many different processes are at play at the same time, including cognitive, social-cognitive and affective. Animal studies in rodents have provided evidence for a neurobiological model called “tag-and-capture”, which postulates that initially weak memories are strengthened through subsequent activation that engages common neural pathways minutes to hours later, through a synaptic mechanism (Frey and Morris, 1997; Ballarini et al., 2009). This model explains how information is selectively consolidated following salient experiences and provides a mechanism by which emotions experienced during learning may influence memory consolidation. This type of learning also exists in humans and it is called emotional learning (Dunsmoor et al., 2015). However, rodent models of emotional learning, while providing direct neurophysiological evidence, use almost exclusively fear conditioning paradigms and tasks requiring navigation or other hippocampus-based information processing. Human models of emotional learning are more diverse in terms of experimental conditioning paradigms and tasks (i.e., investigating different types of memory).

In regards to how emotions influence motor learning, the evidence coming from these studies does not provide a clear picture. For instance, some authors found that emotional learning context did not improve procedural learning (Onal-Hartmann et al., 2012; Gorlick and Maddox, 2015), although it seemed to modulate sequence awareness in an implicit motor sequence learning task (Onal-Hartmann et al., 2012), whereas others found that negative emotional context during initial learning stage enhanced motor memory consolidation after a night of sleep (Javadi et al., 2011). In addition to being scarce, the research on the role of emotional context on procedural learning does not use ecological paradigms and relies on experimental manipulations “borrowed” from animal models (i.e., based on fear conditioning). Nevertheless, this area or research should be expanded to include emotional learning in PD patients given that PD is a neurological condition characterized by both motor and emotional dysfunction due to abnormal activation within the basal ganglia and limbic dopaminergic circuit.

A large number of PD patients have increased levels of anxiety, depression or apathy (McDonald et al., 2003). The emotions generated by these mood states, in interaction with the context in which tasks take place, may drastically affect motor performance and learning. For instance, there is evidence that higher levels of stress, nervousness or anxiety may impair motor performance (Masters, 1992; Wulf and Weigelt, 1997; Wulf et al., 2012), and these had been shown to have the same detrimental effect on procedural learning in healthy people as in individuals with PD (Chiviacowsky et al., 2012). Moreover, the more the learner is experiencing negative emotional responses during learning, the more extra energy is needed for self-regulation and attention, concurrently reducing learning capacity (Hooyman et al., 2014). The role of emotional context in PD is made very evident through a phenomenon known as paradoxical kinesia, which is a sudden, temporary improvement of motor functions, typically followed after some intense stimuli, for instance in a threatening situation (Glickstein and Stein, 1991; Anzak et al., 2011). This is a clear evidence that emotional context may directly affect motor performance in PD. In recent study, Naugle et al. (2012) demonstrated that presentation of positive emotional stimuli improved gait initiation in PD patients. This study probably provides first evidence that the mechanisms responsible for integration of affective and motor processes remain intact in medicated patients. Interestingly, this paradoxical facilitatory effect on motor movement was also demonstrated in PD patients both in ON and OFF medicated states, as well as in healthy age-matched counterparts, when arousing sounds were paired with visual cues triggering the movements (Anzak et al., 2011). These results indicate that the mechanism underlying this phenomenon might be independent of the disturbed dopaminergic pathways, therefore its exploration deserves attention in future research as a potential novel target for treatment of Parkinsonian symptoms, especially if these results could be replicated with more complex motor actions (e.g., motor sequences).

**Figure 2:**
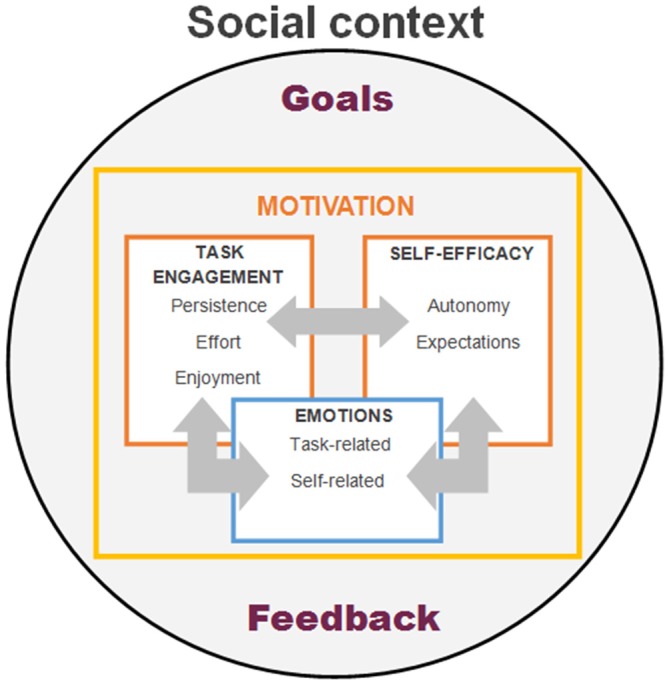
**Schematic model of psychosocial modulators of motor learning and its interactions.** The model proposes that social context may provide both the goals (i.e., social or personal expectations) and feedback (i.e., how well individual’s performance matches the set goals) to the learner. In turn these may affect the interplay between different motivational aspects, such as task engagement, perception of self-efficacy or experienced emotions while learning.

Summing up, more research is needed to investigate how the social context and social interactions may elicit positive or negative emotions, how individuals regulate them during learning and how these may impact individual’s motor learning capacity. In Figure 2 we provide a schema describing our view on how social context may provide goals and feedback to the individual and how these may affect the interplay between different motivational facets. Specifically, we propose that the goals (i.e., which activities to engage in, what tasks to choose from, etc.) arise from the interaction between the individual and his/her social context. Motivational processes include a volitional aspect manifesting as task engagement (i.e., how long to persist and how much effort to exert in the task or activity at hand), which, on the one hand, is shaped by the emotional and self-regulation aspects, and on the other hand, feeds into emotions and self-efficacy based on the feedback received from the task and social context. Exploring and better understanding of these phenomena will help design more effective motor rehabilitation interventions incorporating effective emotion regulation that will help performance not only in PD, but also in other movement disorders.

## Conclusion

In the current work, we provided a brief overview of the psycho-social factors that may affect motor learning in general and its impact in PD, in particular. We argue for the adoption of a more ecological design in future research, closer to real-life situations, and for additional measures that will include the assessment of emotional, motivational states known to affect motor learning and performance. The conclusion of our review is that motor learning research in PD can only benefit from increasing the ecological nature of the context in which tasks are performed, thus augmenting its translational value, because evidence based on artificial laboratory setting may not always generalize to more complex natural environments, including social interaction. Moreover, motor learning in social context has the potential to be used as an intervention strategy to stimulate the motivational compensatory pathways, still intact in early PD, in order to overcome dopamine depletion and associated motor symptoms. For instance, providing positive task emotions and increasing self-efficacy can be used to improve not only motor performance, but also motor learning rates; thus these should be considered important online and offline modulators. However, the most important conclusion for the clinical practice is that each factor that makes motor training more joyful and increases individuals’ motivation and engagement in the task, therefore has the potential to increase patients’ compliance with long term interventions, and prevent further physical decline.

## Author Contributions

Literature search for the study was managed by PZ and OL, first draft of the review was written by PZ, OL and MB critically revised and commented on the manuscript and figures. All the authors substantially contributed to and have approved the final manuscript.

## Conflict of Interest Statement

The authors declare that the research was conducted in the absence of any commercial or financial relationships that could be construed as a potential conflict of interest.
